# Mechanical Force-Induced Color-Variable Luminescence of Carbon Dots in Boric Acid Matrix

**DOI:** 10.3390/molecules28083388

**Published:** 2023-04-12

**Authors:** Shuai Meng, Dengke Cheng, Hailing Gu, Yuchen Li, Yukun Qin, Jing Tan, Qijun Li

**Affiliations:** 1 Institute of Micro-Nano Optoelectronics and Terahertz Technology, School of Mechanical Engineering, Jiangsu University, Zhenjiang 212013, China; 2 Institute for Energy Research, Jiangsu University, Zhenjiang 212013, China; 3 School of Mechanical Engineering, Yangzhou University, Yangzhou 225009, China

**Keywords:** carbon dots, force-induced color-variable luminescence, fluorescence, room temperature phosphorescence

## Abstract

Mechano-luminescent materials that exhibit distinct luminescence responses to force stimuli are urgently anticipated in view of application needs in the fields of sensing, anti-counterfeiting, optoelectronic devices, etc. However, most of the reported materials normally exhibit force-induced changes in luminescent intensity, whereas materials that possess force-induced color-variable luminescence remain rarely reported. Herein, for the first time, a novel mechanical force-induced color-variable luminescence material from carbon dots (CDs) in boric acid (CD@BA) is reported. At low CDs concentration, the luminescence of CD@BA exhibits a grinding-induced color variable from white to blue. This grinding-induced color variable can be switched to yellow-to-white changing by increasing the CDs concentration in BA. The grinding-induced color-variable luminescence originates from dynamic variation in emission ratio of fluorescence and room temperature phosphorescence, due to the influence of oxygen and water vapor in the air. At high CDs concentration, short-wavelength fluorescence undergoes more severe reabsorption compared to room temperature phosphorescence, leading to grinding-induced color-variable switching from white-to-blue to yellow-to-white. Based on the unique properties of CD@BA powder, the applications of recognizing and visualizing fingerprints on the surfaces of various of materials are demonstrated.

## 1. Introduction

Mechanical luminescent color-changing materials can undergo significant luminescent color changing under external mechanical stimuli, such as grinding, cutting, compression, or stretching. Due to the color or intensity of mechanical luminescent materials that can respond to external stimuli, they have good application prospects in clean light sources, mechanical stress sensing, material damage monitoring, anti-counterfeiting, flexible electronics, and other fields [1,2,3,4,5,6,7]. The mechanical luminescent phenomenon was first discovered by Francis Bacon in 1605, where luminescence could be observed by scraping sugar cubes. Tu et al. developed a novel phosphorescent material CaZnOS:Cu in 2015, which exhibits mechanical force luminescence quenching. When a mechanical force is applied to the material, it gradually shows that the departure light is quenched [8]. Xue et al. designed and synthesized nonplanar D-A phenothiazine derivative crystals. The sample after grinding displayed an unprecedented efficient yellow-green room temperature phosphorescence (RTP) enhancement phenomenon (approximately sixfold increase) [9]. However, most of the reported materials normally demonstrated force-induced luminescent intensity changes, whereas materials with force-induced color-variable luminescence still need further development. Liu et al. reported a novel blue-shift and enhanced luminescent material formed by combining energy transfer inhibition and aggregation-induced emission activation [10]. Man et al. developed an organic compound that is highly efficient and undergoes significant changes in green-to-orange fluorescence (FL) at low pressure [11]. The mechanism of mechanical luminescent materials is explained by the changes in the conformation and stacking mode of compounds under mechanical force, such as changes from crystalline to amorphous morphology, or changes between two different crystal phases [12,13]. These materials can control the stacking mode of molecules through grinding, fumigation, and thermal annealing to achieve a reversible conversion of luminescence [14,15]. However, most mechano-luminescent materials are based on organic molecules, organometallic complexes, or organic dye-doped polymers, and these materials usually involve a complex preparation process, high cost, or poor repeatability. Thus, the further exploration of novel mechanical force-induced multi-color luminescent materials with simple synthesis processes, high photo stability, and low toxicity are necessary.

Carbon dots (CDs) are a kind of spherical carbon nanoparticle with luminescence properties and a size of less than 20 nm [16]. As a new class of luminescent nanomaterial, CDs have aroused enormous attention because of their attractive merits such as simple synthetic routes, low cost, low toxicity, and high photo stability [17,18,19,20,21,22,23,24,25,26,27,28,29]. CDs were first discovered at the University of Carolina in the United States in 2004. When purifying single-walled carbon nanotubes from arc discharge ashes, Xu et al. accidentally isolated a carbon nanoparticle with an extremely small size and FL characteristics [30]. The structure of CDs is usually composed of an sp2/sp3 hybrid carbon nucleus, and the surface is usually composed of oxygen/nitrogen-based surface functional groups (-OH, -COOH, -NH_2_) and rich polymer chains [31]. Due to a variety of synthesis methods (top down and bottom up) and a wide range of raw materials (graphite, small molecules, polymers, and even natural materials), various types of FL CDs were rapidly synthesized. For example, Zhang and coworkers synthesized N-doped hydroxy functionalized CDs with an ultra-high quantum yield (QY) of FL 99% [32]. Wang et al. prepared full-spectrum FL emissive CDs that can be further nurtured by slight modifications of the reaction conditions (e.g., temperature, pH) to generate an emission color from blue to red. [33]. In 2013, the first CD-based RTP materials were reported by combining CDs into polyvinyl alcohol (PVA) composite. The obtained films showed a green afterglow after a UV light excitation. The RTP originated from aromatic carbonyls on the CDs surface and the PVA matrix effectively protected their energy from rotational or vibrational loss by rigidifying these groups with hydrogen bonds [34]. After nearly a decade of development, the RTP properties of CDs were greatly improved and developed through various chemical methods, such as heteroatom doping, surface passivation, or surface engineering. Recently, our group exploited a facile aggregation-induced RTP emission strategy from CD-based composites, which exhibited a color fine-tunable and wide-range RTP covering from green to near infrared [35]. Liu et al. controlled CDs@SiO_2_ composite multicolor afterglow emission by adjusting the carbonization degree of CDs at variable calcination temperatures [36]. Lin et al. developed the organic long-lasting luminescence system based on CDs, with an afterglow duration of more than 1 h [37].

Based on the rich optical properties of CDs, a large number of stimulus-response photochromic luminescence CDs have been reported. For example, Li et al. reported that the color changing of RTP from blue to green was achieved by water-induced stimulation of the self-assembly of CDs [38]. Our group achieved alkali-induced color-changing RTP by alkali-induced phenolic hydroxyl ionization on the surfaces of CDs to generate new phosphorescent centers and applied this to information encryption and anti-counterfeiting [39]. Zhao et al. reported CDs with rare extremely acidic sensitivity (PH 1–3). A distinguishable red/brown/green tricolor change was achieved for visual readout and instrument-free sensing by simply tuning the pH to 1, 2, or 3 [40]. Eugenia Kumacheva reported a nanostructured solvent chromic temperature indicator derived from cellulose nanocrystals (CNCs) and CDs. The temperature indicator can observe different FL color responses at different temperatures [41]. However, the development of mechanical force-induced color-variable luminescence in a single CDs system remains a formidable challenge. To the best of our knowledge, force stimuli-responsive CDs materials with dynamic color-changing luminescent properties have not yet been reported.

In this work, a novel mechanical force-induced color-variable luminescence CDs material was developed using the heat treatment of CDs and boric acid (BA) for the first time. At low CDs concentration, the luminous color of the prepared CD@BA changes from white to blue after grinding. This color changing can be switched to yellow-to-white by increasing the concentration of CDs in BA. The grinding-induced color-variable luminescence originates from dynamic variation in the emission ratio of FL and RTP. For the samples with high CDs concentration, the luminescent color of the CD@BA bulk shows a grinding-induced change from yellow to white. The different force-induced color-variable luminescence is attributed to short-wavelength FL undergoing more severe reabsorption at high concentrations than RTP. Moreover, the manufactured CD@BA powder was successfully applied to fingerprint detection and visualization technology, based on unique optical properties.

## 2. Results and Discussion

**Figure 1:**
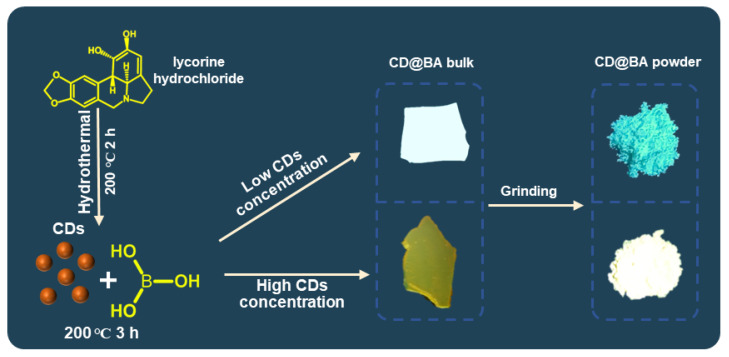
Schematic illustration of the preparation procedure for the CDs and CD@BA bulk and powder.

**Figure 2:**
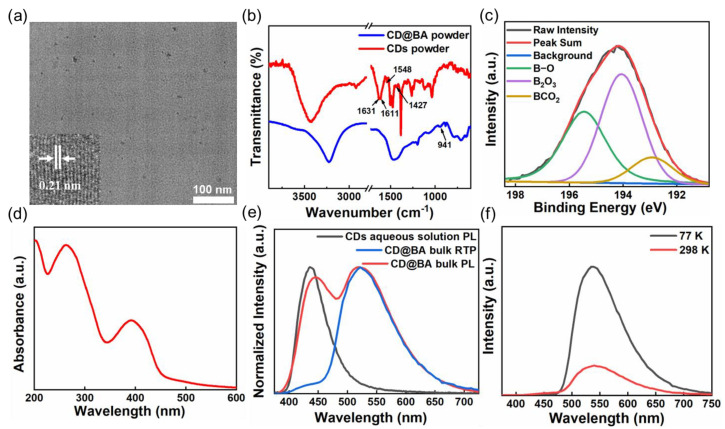
(**a**) TEM image of the CD@BA and inset, corresponding HRTEM image; (**b**) FTIR spectra of CDs and CD@BA powder; (**c**) High-resolution B 1 s XPS spectrum of CD@BA powder; (**d**) UV-visible absorption spectrum of CDs aqueous solution; (**e**) PL emission spectrum of the CDs aqueous solution, PL and RTP emission spectra of the CD@BA bulk; (**f**) RTP emission spectra of CD@BA bulk at 298 and 77 K, under 365 nm excitation.

The preparation of CDs and the corresponding composite with BA is summarized simply in Figure 1. In detail, the CDs were synthesized by a one-pot hydrothermal treatment of lycorine hydrochloride at 200 °C for 2 h, whereas CD@BA bulk was prepared facilely via a thermal treatment of a mixture of CDs and BA. The morphologies of the CDs in CD@BA were analyzed using TEM and HRTEM (Figure 2a). Figure 2a shows that the CDs had an average diameter of 4.3 ± 1.2 nm and a well-resolved lattice spacing of 0.21 nm, corresponding to the (100) facet of graphite carbon. 

The chemical structure and photophysical properties of CDs and CD@BA were studied. The structure and composition of CDs and CD@BA powders were investigated using the FTIR spectrum (Figure 2b). The FTIR spectrum of CDs exhibits characteristic absorption peaks at 3141–3772, 1631, and 1611 cm^−1^, which were assigned to the O-H, C=O, and C=C stretching vibrations, respectively. The characteristic absorption peaks located at 1429 and 1548 cm^−1^ demonstrated the existence of C-N and C=N on CDs. After incorporating CDs into the thermally annealed BA matrix, a new absorption peak can be observed at 941 cm^−1^, which corresponds to the C-B stretching vibration, indicating BA molecules functionalized on the CDs surface after thermal treatment [42,43,44,45,46]. To further illustrate the chemical structure, chemical bonds were analyzed using XPS. As shown in Figure 2b, the XPS results of CD@BA, the high-resolution B 1s spectrum of CD@BA can be fitted to three bands centered at 195.3, 193.9, and 192.9 eV, which belong to B-O, B_2_O_3_, and BCO_2_, respectively [47], meaning a dehydration process occurs between CDs and BA. Therefore, the XPS and FTIR analyses indicated that CDs are successfully embedded in BA matrix and covalent coupling reactions occur between CDs and BA.

The optical characteristics of CDs were investigated by measuring the absorption, which was collected in Figure 2d. The UV-visible absorption spectrum of CDs aqueous solution shows two dominating absorption bands at approximately 265 and 390 nm, which are attributed to the π-π* transition of C=C bonds and n-π* transition of C-N/C=N bonds, respectively [48,49]. As shown in Figure S1, the excitation spectrum of CD@BA bulk under 530 nm emission, the excitation bands located in the range from 300 to 500 nm overlap with the absorption band of C-N/C=N [50]. Therefore, the above results show that the RTP emission band at 530 nm mainly originates from the C-N/C=N bonds in CDs. 

Under daylight, the CD@BA bulk appears transparent with a light-yellow color (Figure 1 and Figure S2); the CD@BA bulk exhibits yellow-green PL under UV lamp excitation (365 nm), and after the UV lamp is switched off, the yellow RTP can last for about 9 s under natural conditions. In Figure 2e, the PL spectrum of CD@BA bulk displays two significant emission peaks approximately at 430 and 530 nm. The emission peak at 530 nm in the PL spectrum of CD@BA bulk almost coincides with its peak in the RTP spectrum. Moreover, the CDs aqueous solution contains a main emission peak at 430 nm, which almost coincides with the emission peak at 430 nm in the PL spectrum of the CD@BA. It is well known that the quenching of RTP usually occurs under water due to the strong hydrogen bond-breaking ability of water molecules. Therefore, the peak at 530 nm is mainly derived from RTP. In addition, the temperature-dependent RTP spectrum of CD@BA bulk (Figure 2f) shows that the RTP emission intensity 530 nm is significantly increased at 77 K compared to 298 K. The temperature-dependent phosphorescent decay spectrum of CD@BA bulk (Figure S3) reveals that the phosphorescent delay lifetime increased at 77 K compared to 298 K. The FL decay spectrum of CD@BA bulk (Figure S3b) indicates a lifetime of 12.2 ns in the 430 nm band, confirming its fluorescence nature. Based on the above analysis, it is further indicated that the peak at 530 nm in the PL spectrum is mainly derived from RTP rather than delayed FL. 

**Figure 3:**
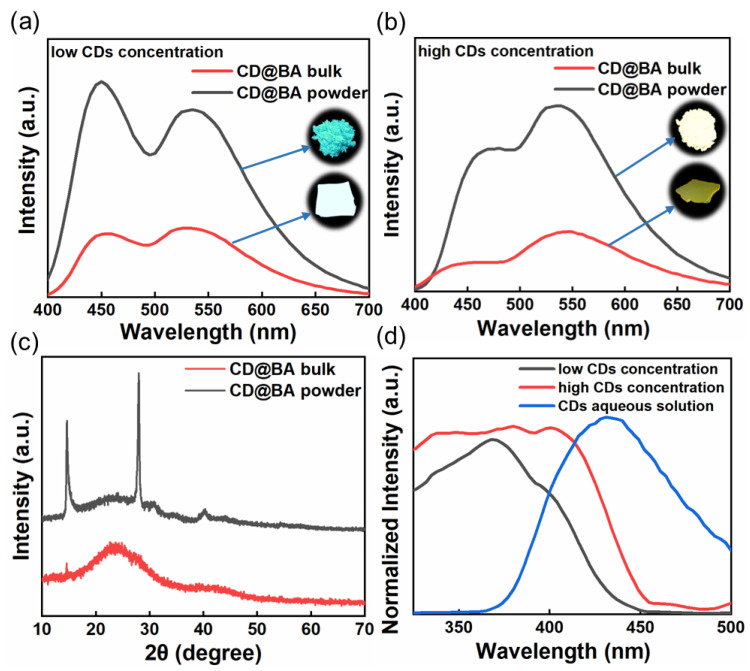
(**a**) PL emission spectra and digital picture of the CD@BA bulk and CD@BA powder at low CDs concentration under 365 nm lamp; (**b**) PL emission spectra and digital picture of the CD@BA bulk and CD@BA powder at high CDs concentration under 365 nm lamp; (**c**) XRD spectra of the CD@BA bulk and CD@BA powder; (**d**) The excitation spectra (λ_em_ = 530 nm) of CD@BA with different CDs concentrations and FL emission spectrum of the CDs aqueous solution (blue line).

In further experiments, we prepared CD@BA bulk with different concentrations of CDs and studied their normalized PL spectra under 365 nm excitation (Figure S4, Table S1). Interestingly, the CD@BA displays an unprecedented luminescent color-changing phenomenon before and after grinding. As displayed in Figure 3a, the luminescence color of CD@BA with low CDs concentration exhibits white-to-blue transition after grinding. The grinding-induced color variable can be switched to yellow-to-white changing at high CDs concentration (Figure 3b). The PL emission spectra of CD@BA with different CDs concentrations (Figure 3a,b) show that both the yellow RTP and blue FL intensities enhance after grinding, but they exhibit different enhancement ratios. The XRD spectrum (Figure 3c) for the CD@BA bulk presents broad and weak peaks, whereas the CD@BA powder presents several sharp diffraction peaks, indicating a grinding-induced structural transition from an amorphous to a crystal phase. A more rigid crystalline environment can effectively reduce nonradiative transitions compared to amorphous environments, resulting in FL and RTP enhancement [51,52,53]. Before and after grinding, the FL enhancement is greater than RTP. This is mainly because the RTP of the fine-grained particles after grinding is more sensitive to oxygen and water vapor in the air. We tested the PL emission spectra of CD@BA powder stored in air and in a vacuum environment (Figure S5). Under air conditions, the decrease in the RTP of CD@BA powder is greater than that of the FL, whereas there is almost no quenching when CD@BA powder is stored in a vacuum environment. These results show that RTP is more sensitive to oxygen and water vapor in the air. However, the fine particles produced by grinding make the RTP of CDs more easily quenched by oxygen and water vapor in the air, resulting in the increase of FL and RTP intensity in different proportions. Therefore, a grinding-induced luminous color blue-shift from CD@BA was observed. The normalized RTP excitation spectra of CD@BA with different CDs concentrations (Figure 3d) show that the RTP excitation spectrum with an emission at 530 nm overlaps with its FL emission peak and the overlap area is larger at high CDs concentration. Therefore, we concluded that the different grinding-induced color-variable luminescence is mainly attributed to the short-wavelength FL, which undergoes more severe reabsorption at high CDs concentrations, leading to the intensity of FL being lower than that of phosphorescence.

**Figure 4:**
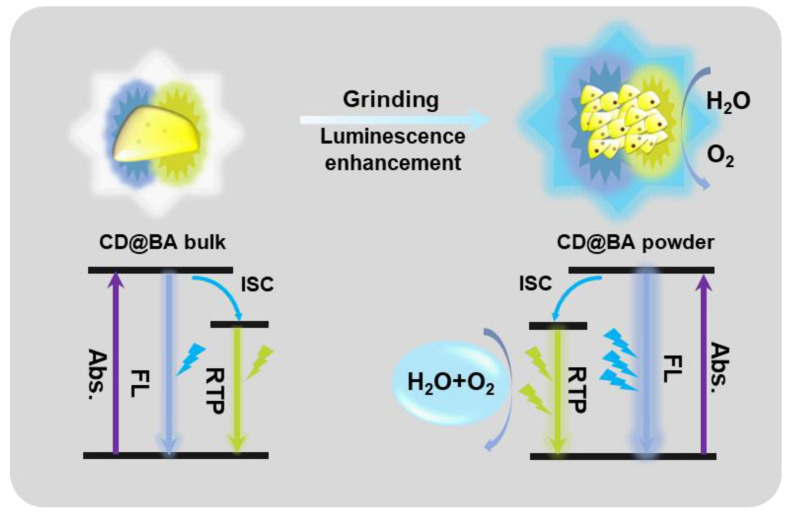
Schematic of the proposed force-induced color-variable luminescence mechanism of CD@BA composites. Abs., absorption; FL., fluorescence; RTP, room temperature phosphorescence.

Based on the above results, we propose a force-induced color-variable luminescence mechanism according to the schematic illustration presented in Figure 4. After grinding, the transition of CD@BA from amorphous to crystalline state occurs instantaneously, leading to a significant enhancement in both FL and RTP emission intensity. However, because the RTP of the fine-grained particles after grinding is more sensitive to oxygen and water vapor in the air, the increase of FL and RTP intensity occurs in different proportions. Thus, force-induced color-variable luminescence is realized. The observed variations in luminescence color for CD@BA at high CDs concentration are primarily attributed to the short-wavelength FL undergoing more severe reabsorption at high CDs concentration.

**Figure 5:**
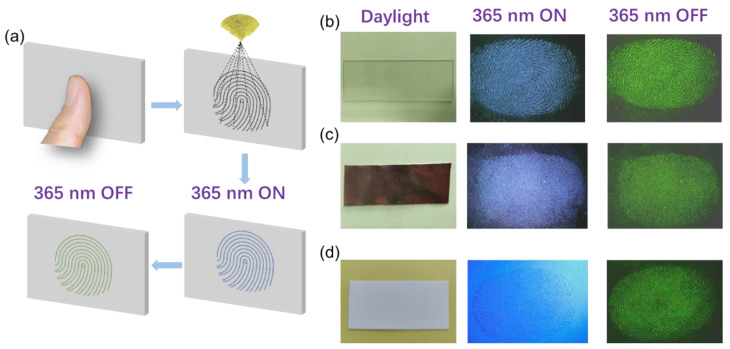
(**a**) fingerprint identification process of fingerprint morphology; (**b**) transparent, (**c**) opaque, and (**d**) fluorescent materials under different light conditions.

CD@BA powder has promising potential in fingerprint detection technology; it successfully detects and visualizes fingerprints from different materials due to its advantageous properties. As shown in the fingerprint identification process of fingerprint morphology in Figure 5a, fingerprints are pressed onto the surfaces of various materials, then CD@BA powder is sprayed onto the fingerprint area. The area with fingerprints absorbs a large amount of powder and shows blue FL under the UV lamp. The green RTP appears after the UV lamp is turned off. For different materials, fingerprints can be detected using the luminescence of the CD@BA powder before or after irradiation using UV lamps. For example, Figure 5b,c show that the fingerprint is easily visible on non-fluorescent substrate materials under a UV lamp. However, in Figure 5d, the substrate material is fluorescent, and the fingerprint is difficult to distinguish when the UV lamp is turned on. However, after the UV lamp is turned off, the area with fingerprint traces will still exist RTP. Because the substrate materials have no RTP, the fingerprint remains clearly visible after the UV lamp is turned off. Therefore, CD@BA powder can effectively extract fingerprints on the surface of various materials. 

## 3. Materials and Methods

### 3.1. Chemicals and Reagents

Unless otherwise noted, all reagents were purchased from chemical reagent suppliers and can be used without further purification. Lycorine hydrochloride (AR, 5 g) and boric acid (99.5%, 500 g) were purchased from Aladdin (Shanghai, China). Deionized (DI) water (18.2 MΩ cm at 25 °C), obtained using a Milli-Q (MQ) water system, was used in all the experiments.

### 3.2. Apparatus

The transmission electron microscope (TEM) and high-resolution transmission electron microscope (HRTEM) images were acquired using a Talos F200X (FEI, Hillsboro, OR, USA) TEM operating at 200 kV. TEM is a type of accelerated and concentrated electron beam transmitted onto a very thin sample; electrons collide with atoms in the sample and change direction, resulting in stereo-angle scattering. The size of the scattering angle is related to the density, thickness, etc., of the sample, so it is possible to form a light and dark image, and the image is magnified, focused, and displayed on imaging devices (such as phosphor screens, films, and photosensitive coupling components). For TEM measurements, a drop of solution was placed on a carbon coated copper mesh and allowed to dry naturally before observation. 

Fourier transform infrared (FTIR) spectroscopy was performed on a Bruker Tensor 27 spectrophotometer (Bruker, Billerica, MA, USA); the scanning wavenumber range was 4000–400 cm^−1^, and the accuracy was 0.01/2000 cm^−1^. When infrared light with a continuous wavelength passes through the substance, the vibration frequency or rotation frequency of a group in the molecule of the substance is the same as the frequency of infrared light, the molecule absorbs energy from the original ground state oscillation (rotation) kinetic energy level to a higher energy vibration energy level, the molecule absorbs infrared radiation after vibration and rotation energy level transition, and the wavelength of light is absorbed by the substance. Therefore, infrared spectroscopy is an analytical method to determine the molecular structure of substances and identify compounds based on the relative vibration between atoms and molecular rotation inside molecules.

UV-visible absorption spectroscopy was performed using a Shimadzu UV-3600 spectrophotometer (Shimadzu, Kyoto, Japan) and collected in the range of 200–800 nm, with a step size of 0.5 nm and 0.5 sec/step. UV-visible absorption spectroscopy is caused by the transition of valence electrons after molecules (or ions) absorb ultraviolet or visible light. Because transitions between electron energy levels are always accompanied by transitions between vibration and rotational energy levels, the UV/VIS spectrum exhibits a wide band.

X-ray photoelectron spectroscopy (XPS) measurements were performed using an ESCALAB 250 XPS system (Thermo Electron Corporation, Waltham, MA, USA). Based on photoionization, when the energy of the excitation source is fixed, the energy of its photoelectrons is only related to the type of element and the atomic orbital of the ionization excitation, so that the element type of the substance is qualitatively analyzed according to the binding energy of the photoelectrons. After X-ray irradiation, the intensity of the photoelectrons emitted from the sample surface has a linear relationship with the concentration of the atom in the sample, and further semi-quantitative analysis of the elements can be performed. In addition, an important application of XPS is the analysis of the chemical valence state of elements. It can be used to study the elemental type, chemical valence, and relative content of the sample surface (1–10 nm) of various solid materials. 

X-ray diffraction (XRD) patterns were obtained with a Rigaku 18 KW D/max-2550 (Rigaku, Tokyo, Japan) using Cu-Ka radiation and collected in the 2θ range of 10–70°. It can be used for crystal structure analysis, structure refinement, phase analysis and multi-phase content analysis of inorganic materials, crystallinity analysis of polymer materials, and can be analyzed and tested for powder, block, and thin film samples.

Time-resolved RTP decay curves were tested by the time-correlated single-photon counting (TCSPC) technique on an FS5 FL spectrophotometer with a PS pulsed laser of 365 nm. The PL, absolute QYs, and RTP lifetimes were measured using an Edinburgh FS5 spectrophotometer (Edinburgh, Edinburgh, UK) equipped with a xenon arc lamp (Xe 900), microsecond flashlamp (µF 900), and an integrating sphere. The luminescence lifetimes (τ) were obtained by fitting the decay curve with the following multi-exponential decay function:

(1) $$I\left(t\right)=\sum_{i}A_{i}e^{-\frac{t}{\tau_{i}}},$$

where *A_i_* and *τ_i_* are the amplitude and lifetime of the individual components of the multi-exponential decay profiles, respectively. 

The photographs were taken using an intelligent mobile phone in different environments (daylight and 365 nm).

### 3.3. Synthesis of CDs

The CDs were synthesized using a simple hydrothermal method. Typically, lycorine hydrochloride (0.1 g) and deionized water (30 mL) were added to a Teflon-lined autoclave (100 mL) and sonicated for 1 min to ensure a homogenous mixture. Then, the equipment was used in a high-temperature blast drying oven (BPG-9050AH, Yiheng Technology, Shanghai, China) heated at 200 °C for 2 h. After completion of the hydrothermal carbonization process, a light-yellow dispersion solution was obtained. The resulting light-yellow solution was centrifuged at high speed (8000 rad/min) for 5 min to remove large or condensed particles, and the above steps were repeated three times. The solution was then dialyzed with DI water using a dialysis membrane (500 Da) for 12 h; the water was changed three times during this period. Then, the dispersions were frozen at −18 °C for 24 h and set sequentially to be lyophilized for 72 h. Again, this produced brown powder products. This lyophilized CDs powder was then used for research. 

### 3.4. Synthesis of CD@BA with Different Concentration of CDs

Boric acid (3 g) was first diluted with DI water (30 mL) and mixed with the CDs (2, 13, 20, 50, 100, 150, and 200 mg) in a beaker. The beaker was sonicated for 20 min to completely dissolve the BA. Next, the beaker was covered by perforated aluminum foil, placed in an oven at 200 °C for 3 h, and subsequently cooled down naturally to room temperature to form glassy state CD@BA bulk. Finally, CD@BAs with different CDs concentrations were obtained. Low CDs concentrations and high CDs concentrations were prepared using 13 mg and 200 mg of CDs solution, respectively. 

## 4. Conclusions

In summary, we developed a novel CD@BA composite that exhibits grinding-induced color-variable luminescence. The as-prepared CD@BAs with low and high CDs concentrations exhibit a grinding-induced color-variable luminescence from white to blue and yellow to white, respectively. Further studies showed that blue FL increases more significantly than RTP after grinding; it may be that the RTP of the finely grained particles after grinding is more easily quenched by oxygen and water vapor in the air. At a high CDs concentration, short-wavelength FL undergoes more severe reabsorption compared to RTP, leading to luminescence color changes from yellow to white. In addition, we demonstrated the application to fingerprint detection and visualization technology by using the unique luminescent properties of CD@BA powder. This work not only provides a convenient method for the manufacture of grind-induced color-variable luminescent materials, but also paves the way for the design and construction of next-generation stimulus-responsive CDs materials. 

## Data Availability

The data presented in this study are available within the article and supplementary material.

## Supplementary Materials

The following supporting information can be downloaded at: https://www.mdpi.com/article/10.3390/molecules28083388/s1, Figure S1: Phosphorescence excitation spectrum of CD@BA bulk under 530 nm emission; Figure S2: Digital photographs of CD@BA bulk before and after turning off the 365 nm UV-irradiation source and under daylight conditions; Figure S3: Room temperature phosphorescence decay curves of CD@BA bulk at 77 k and 298 k (λex = 365 nm and λem = 530 nm); Figure S4: The normalized PL emission spectra of CD@BA bulk with different CDs concentrations under 365 nm lamp; from top to bottom: 2, 13, 50, 100, 150, 200 and 250 mg; Figure S5: PL emission spectra of CD@BA powder stored in air and vacuum environment, respectively; Table S1: emission spectral data of CD@BA with different CDs concentration.
