# Supplementary material for: Mechanical Force-Induced Color-Variable Luminescence of Carbon Dots in Boric Acid Matrix
Source: Molecules. 2023 Apr 12;28(8):3388. doi: 10.3390/molecules28083388 (PMC10141381; doi:10.3390/molecules28083388)
Supplement: Supplementary file 1 [file molecules-28-03388-s001.zip › molecules-2321091-supplementary.pdf]

Supporting Information

Mechanical Force-Induced Color-Variable Luminescence of Carbon Dots in Boric Acid Matrix

Shuai Meng [1†], Dengke Cheng [1†], Hailing Gu [1],Yuchen Li [1], Yukun Qin [2], and Jing Tan [1,*], Qijun Li [3,*]

[1] Institute of Micro-nano Optoelectronics and Terahertz Technology, School of Mechanical Engineering, Jiangsu University, Zhenjiang 212013, China

[2] Institute for Energy Research, Jiangsu University, Zhenjiang 212013, China

[3] School of Mechanical Engineering, Yangzhou University, Yangzhou 225009, China

[*] Correspondence: tanjing@ujs.edu.cn; liqijun@yzu.edu.cn

† These authors contributed equally to this work.

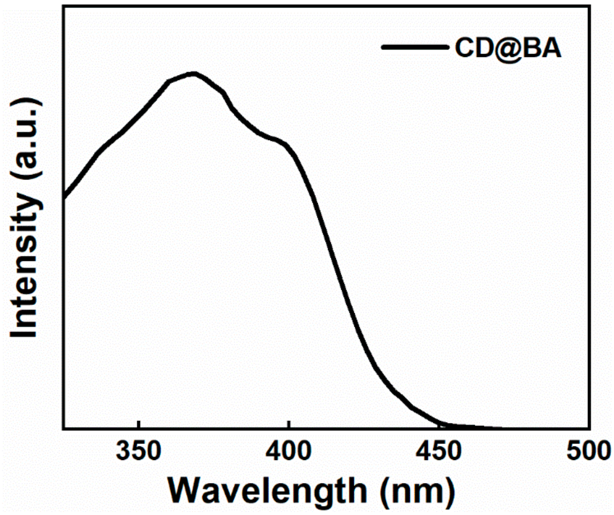

Figure S1 Phosphorescence excitation spectrum of CD@BA bulk under 530 nm emission.

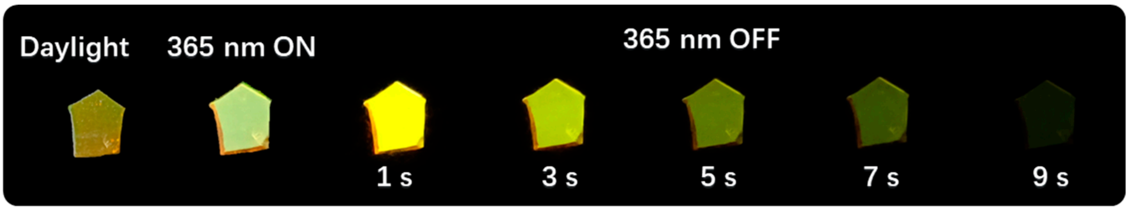

Figure S2 Digital photographs of CD@BA bulk before and after turning off the 365 nm UV-irradiation source and under daylight conditions.

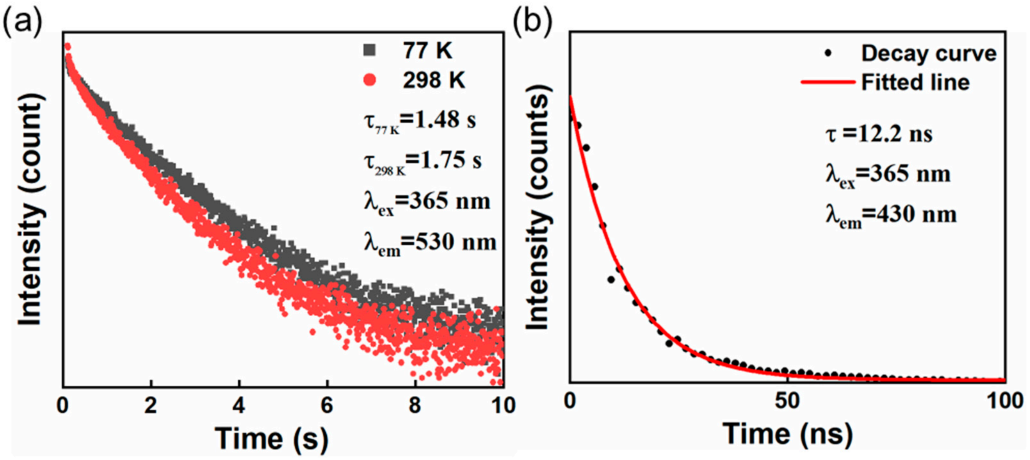

Figure S3 (a) Room temperature phosphorescence decay curves of CD@BA bulk at 77k and 298k respectively. (λex = 365 nm and λem = 530 nm); (b) FL decay curve of CD@BA bulk.

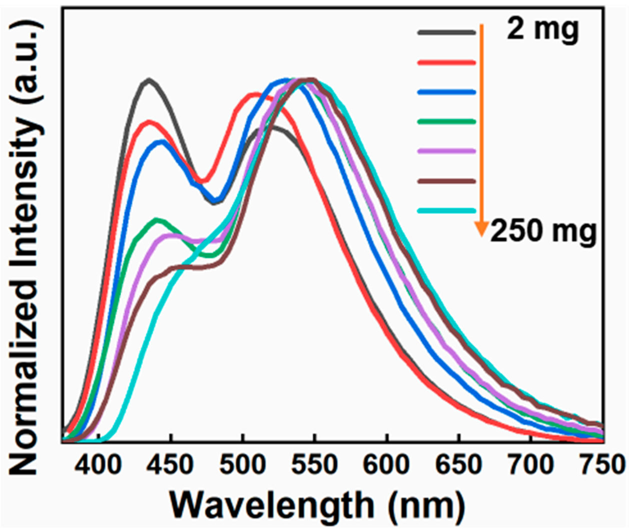

Figure S4 The normalized PL emission spectra of CD@BA bulk with different CDs concentrations under 365 nm lamp; from top to bottom: 2, 13, 50, 100, 150, 200 and 250 mg.

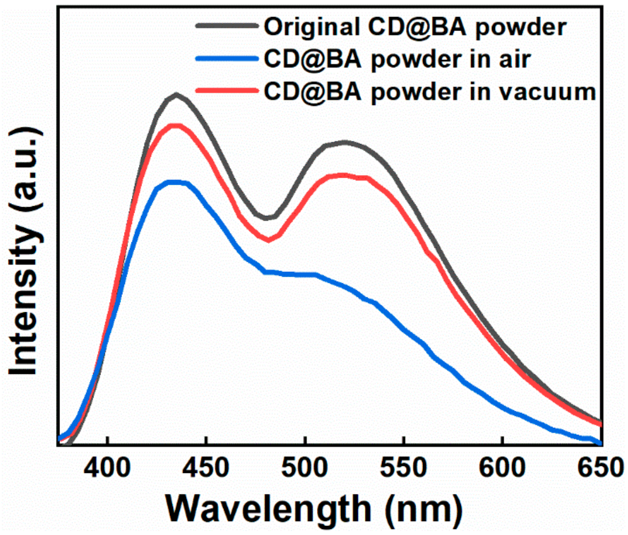

Figure S5 PL emission spectra of CD@BA powder stored in air and vacuum environment, respectively.

Table S1 emission spectral data of CD@BA with different CDs concentration

Serial number	CDs concentration (mg)	Fluorescence peak position (nm)	Phosphorescence peak position (nm)
**1**	2	428	520
**2**	13	430	520
**3**	50	440	530
**4**	100	440	535
**5**	150	450	540
**6**	200	450	545
**7**	250	450	545
